# AFOROS: A Low-Cost Wi-Fi-Based Monitoring System for Estimating Occupancy of Public Spaces

**DOI:** 10.3390/s21113863

**Published:** 2021-06-03

**Authors:** Mario Vega-Barbas, Manuel Álvarez-Campana, Diego Rivera, Mario Sanz, Julio Berrocal

**Affiliations:** ETSI de Telecomunicación, Universidad Politécnica de Madrid (UPM), Avda. Complutense 30, 28040 Madrid, Spain; manuel.alvarez-campana@upm.es (M.Á.-C.); diego.rivera@upm.es (D.R.); mario.sanz@upm.es (M.S.); julio.berrocal@upm.es (J.B.)

**Keywords:** Wi-Fi tracking, automatic people counting, occupancy estimation, passive tracking, Wi-Fi sensor, Wi-Fi probe

## Abstract

Estimating the number of people present in a given venue in real-time is extremely useful from a security, management, and resource optimization perspective. This article presents the architecture of a system based on the use of Wi-Fi sensor devices that allows estimating, almost in real-time, the number of people attending an event that is taking place in a venue. The estimate is based on the analysis of the “probe request” messages periodically transmitted by smartphones to determine the existence of Wi-Fi access points in the vicinity. The method considers the MAC address randomization mechanisms introduced in recent years in smartphones, which prevents the estimation of the number of devices by simply counting different MAC addresses. To solve this difficulty, our Wi-Fi sensors analyze other fields present in the header of the IEEE 802.11 frames, the information elements, to extract a unique fingerprint from each smartphone. The designed system was tested in a set of real scenarios, obtaining an estimate of attendance at different public events with an accuracy close to 95%.

## 1. Introduction

Tracking technologies applied to specific locations or enclosures allow a quick and efficient response to different problems associated with controlling capacity or occupancy.

From the point of view of civilian protection or state and armed forces, it is difficult to obtain accurate data in real-time regarding the capacity of a location or space. Not handling this type of information dynamically increases the risk of possible accidents due to exceeding the maximum occupancy allowed [1,2]. Currently, all municipalities and states have laws that regulate the maximum capacity and occupancy of open and enclosed spaces devoted to entertainment, i.e., shopping centers, theaters, concert halls, etc., or in public transport. Specifically, given the current situation caused by COVID-19, having this type of information takes on particular relevance since these regulations and restrictions are constantly modified depending on how the pandemic evolves [3].

In physical security, it is crucial to ensure no presence in specific zones or restricted areas. Generally, physical security systems are static systems installed at the entrance to an enclosure or distributed within site, providing different levels of security [4,5]. The problem with this type of system is that, if trespassed, it makes it relatively difficult to identify unauthorized activity in a restricted area.

From the point of view of optimizing civil infrastructures, real-time data, if possible, or near real-time in other cases, are necessary to manage available resources efficiently. For example, the management of the public transport network may be underutilized if its management is based on historical data rather than real-time data related to occupancy [6,7,8]. In addition, there is a great challenge related to the estimation models of movement and occupation in urban environments, mainly since these types of models are usually fed with artificial data and patterns, which do not accurately reflect the natural behavior of the target to model [9].

Based on the above, the need to develop a cost-efficient solution based on people tracking arises. The solution should be used by personnel without technical knowledge, with simple implementation, and with accuracy in the detected occupancy results.

In this sense, although there exist different technologies that allow tracking or counting, this research focused on using Wi-Fi technology and smartphones. The cheapening of smartphones and their usefulness have favored their inclusion in people’s daily lives [10,11]. For our purposes, they are interesting because of the omnipresence of Wi-Fi interfaces in them. These devices allow continuous connection to the internet and offer a trail of connectivity that allows the monitoring and control of their presence. Even though this could be interpreted as a vulnerability of the people’s freedom, it has been verified that the tracking of devices in exceptional situations (pandemics, terrorist attacks, etc.) favors the safeguarding of general security [12]. In any case, through anonymization techniques, such as those carried out by non-contact sensing and tracking systems for COVID-19, it is possible to guarantee people’s privacy [13].

Thus, this paper presents a system that enables precise assessments of the occupancy of an indoor or outdoor environment by using passive, non-intrusive, and low-cost Wi-Fi sensors. Its generic design approach allows its application to other kinds of mass events or large public spaces, e.g., sports venues, shopping centers, protests, airports, and other civil infrastructures.

The rest of the article is organized as follows to clarify the development and achievement of the main goal of this research. First, Section 2 presents the background of the research that supports the development presented. Then, Section 3 points out the system specification and technical requirements. Section 4 provides an overview of the system architecture and a description of each underlying subsystem’s design and implementation details. For its part, the adjustment and validation tests of the platform are presented in Section 5. Finally, Section 6 discusses and summarizes the main contributions of the research work.

## 2. Background

As previously introduced, this work focused on three essential concepts in people tracking: the type of interaction between the tracking system and the tracked devices, Wi-Fi-based monitoring, and MAC address randomization. These concepts represent the background of the research work performed and are analyzed in the following subsections.

### 2.1. Active and Passive Tracking Systems

Currently, some systems and technologies allow people to be tracked and then control the occupancy of certain environments. In this article, we differentiate technologies depending on the needs for interaction with the owner or device being counted or monitored. Following that, we can classify technologies into two main groups: active systems and passive systems.

#### 2.1.1. Active Systems

This kind of system uses technology that requires specific actions by the owner of the device being monitored (e.g., app installation, granting of permissions, etc.). In general, this kind of system relies on technologies such as Bluetooth [14] and geolocation [15].

Systems based on Bluetooth analyze Bluetooth traffic emitted by smart devices. Generally, in active systems, the device must be broadcasting its identifier to be counted. In applications such as Radar COVID [16] that do not necessarily identify the device, a prior installation by the device owner is also required anyway.

In geolocation-based systems, there have been developed applications that allow the location of devices to be reported using the GPS module available at the terminals. An example of this type of application that uses this technology to detect agglomerations is Google Maps (focused on traffic jams). In this case, it is necessary to have the GPS features turned on, and the device owner must enable permissions in the navigation application to be used.

#### 2.1.2. Passive Systems

In this type of system, technologies that do not require device owners to carry out specific actions are used. In fact, in most cases, their operation is transparent for them.

Examples of this type of system are those based on infrared beams [17]. They use physical equipment with an infrared beam at the single entrance to a space, counting the number of interruptions to determine the number of people who have entered or left said space. Another set of passive systems are those based on reconnaissance cameras [18]. This system is based on capturing images in real-time and, using visual recognition techniques, counting the number of detections of faces, bodies, or other parts of the human body. Moreover, cellular technology or mobile telephone/data networks allow passive tracking systems [19,20]. In these systems, an analysis of the device’s presence in the radius of action of the antennas to which the terminal is connected is carried out. There exist systems that allow the detection of devices in 2G, 3G, and 4G networks [21,22]. For its part, Wi-Fi and Bluetooth technology also offer an interesting opportunity for developing passive tracking systems [23]. Although in this article we only focus on Wi-Fi technology, in both cases, it is possible to use devices capable of analyzing probe request messages issued by any smartwatch device, smartphone, etc. Finally, there are developments of sensors and specific devices for tracking people in crowded environments [24]. These are hardware specialized in tracking people in different environments (public transport, large stores, etc.). These systems are designed completely focused on the end-use case.

Even though all the exposed technologies meet the requirements to carry out capacity control, they present different advantages and disadvantages. Many of them, such as systems based on infrared beams, cellular technologies, or systems based on recognition cameras, imply a high cost in infrastructure for sensorization and information processing. On the other hand, the solution presented in this article aims to make use of a technology that allows obtaining accurate estimations while maintaining low costs and presents simplicity of operation. In addition to these reasons, our proposal aims for scenarios where there is a need for passive tracking, which implies using a technology that does not require specific actions by the device owner to perform face-to-face detection. Due to the requirements set forth and taking advantage of the growing trend in using Wi-Fi technology from smartphone devices, we selected Wi-Fi wireless communication technology as the base for our system.

### 2.2. Wi-Fi-Based Monitoring

The increase in the use of smartphone devices invites us to consider monitoring solutions based on the technologies present in these types of phones, e.g., Wi-Fi, Bluetooth, GPS, etc. Passive monitoring of Wi-Fi devices represents an interesting low-cost solution for counting people, valid both in indoor and outdoor scenarios [25,26,27,28].

A large amount of the work in this area has focused on tracking people, both in open and closed spaces and with very different objectives, i.e., marketing, security, etc. [25,26,27,28]. These works are not as interested in counting people as offering models of human behavior that can be used for specific purposes. Because this modeling is a complex task, most of these research works pointed out the need to install certain infrastructure in the spaces to be monitored, raising the cost of the solution, reducing its reuse, or even adding privacy implications.

The works closest to our research are those that focus on counting people in spaces and that leave aside the analysis of the behavior of the people counted. These solutions, in general, are classified into two groups, those that analyze the Wi-Fi signal [29,30,31,32] and those that focus on the analysis of the information packets sent by the Wi-Fi devices [33,34,35].

Solutions based on Wi-Fi signal analysis (the received signal strength indicator or RSSI and the channel state or CSI, above all) show promising results but for in indoor setups. Works such as [31,32] performed laboratory tests for highly controlled environments, with no results in open or uncontrolled spaces. Other research based on the analysis of Wi-Fi signals, such as [30] required the installation of firmware in the access points (APs).

On the other hand, solutions based on the analysis of probe requests do offer experimentation in uncontrolled and open spaces [33,34,35]. These solutions apply to indoor and outdoor spaces and are a general-purpose approach, facilitating their reuse in different contexts. However, the solutions analyzed present shortcomings when it comes to tackling the problem of randomization of MAC addresses, which is discussed in the next subsection.

From the point of view of the accuracy of the results, regardless of the approach used, the necessary infrastructure, or the type of experimentation, the analyzed articles that were used as a basis for our research show very different values, between 80% and 96% [29,30,35]. These values can only be considered for direct comparison with special caution, as they come from developments based on very different experimentation approaches.

### 2.3. MAC Address Anonymization

MAC addresses are sent in the clear during the exchange of communication packets between devices. However, for some time, mobile technology manufacturers have intensified their efforts to promote and enforce device owner privacy, trying to protect Wi-Fi communications by anonymizing MAC addresses. Such anonymization techniques are aimed at avoiding using the device’s real MAC address (a random fake one is used instead) until a connection to a Wi-Fi network is accomplished.

This solution depends on the manufacturer and the operating system [36] since no standard regulates this type of anonymization technique. For example, in the case of iOS, the solution involves sending MAC addresses managed locally by the device in the probe frames, randomly selecting the three least significant bytes of the MAC address. In the case of Android, some smartphone manufacturers have decided to use random MAC addresses in the probe frames corresponding to the MAC address ranges assigned by the IEEE.

From the University of Hasselt (Belgium), an approach based on Raspberry Pi equipment was developed and detailed in [37]. They tried to solve the same problem raised in our research work. As in our case, their solution made use of Wi-Fi tracking technology, analyzing probe requests and developing a low-cost, functional Wi-Fi sensor. Even though the results presented in the validation show good results, and mainly due to the evolution of technology, today’s factors that complicate the detection of devices were not taken into account. Factors such as the inconsistency of sensorized data in the same space by part of various sensors and the extraction of information from the frames with random MAC addresses were not addressed in that solution.

Other newer solutions use machine learning models to solve the problem of MAC address randomization [38,39]. In both cases, they present promising results for real test scenarios. However, these scenarios lack a large number of people, so it is not possible to determine the operation of these proposed solutions in a highly crowded environment, as is the focus of our research.

## 3. System Specification

As explained in Section 1, the aim of AFOROS is to facilitate the intelligent control of the capacity of an environment destined to host massive or highly crowded events, especially outdoors, e.g., music festivals and large concerts. The business cases for AFOROS include public and private entities in charge of safeguarding people’s safety at the local level, as well as those in charge of collecting copyright or taxes. Therefore, the platform must be affordable in terms of cost, i.e., low-cost hardware components, and easy to use. Moreover, it must support real-time data, or near real-time, and, if it is not possible, it must support operations to ensure full awareness of the analyzed situation and an early response if necessary.

Since the main objective of the platform is focused on counting people, the security and privacy of the data collected and generated is a very important requirement. Thus, accredited access and use of the platform should be ensured, according to the operator’s role and granted permissions, as well as the integrity and confidentiality of the data received and sent to and by the system. In addition, the anonymization techniques and the rest of the security features should provide the possibility for compliance with information and data privacy or data protection regulations, e.g., the General Data Protection Regulation (GDPR) [40].

From the point of view of the minimum precision required of the system, when estimating the number of people accounted for, it is not easy to establish an exact value. In the case of Spain, the statistics indicate penetration of mobile telephony in households of 99.5%, while 95% of internet users (97% of the population between 16 and 56 years of age in Spain) state that they have used the mobile phone to access it from outside the home [41]. This determines that around 92.15% will carry a device. Therefore, this could be seen as a maximum error value for the estimations that our proposal is able to make. If the counting value presents an error above 7.85%, we are probably counting too many or too few people. Obviously, this threshold is also an estimation and, therefore, should be treated with caution.

Finally, the need to cover different kinds of events and environments suggests that the platform should be as scalable and flexible as possible, making it possible to update the hardware and software when necessary. This way, the design and implementation of the platform should be based on standardized and open solutions.

## 4. Proposed Architecture

**Figure 1:**
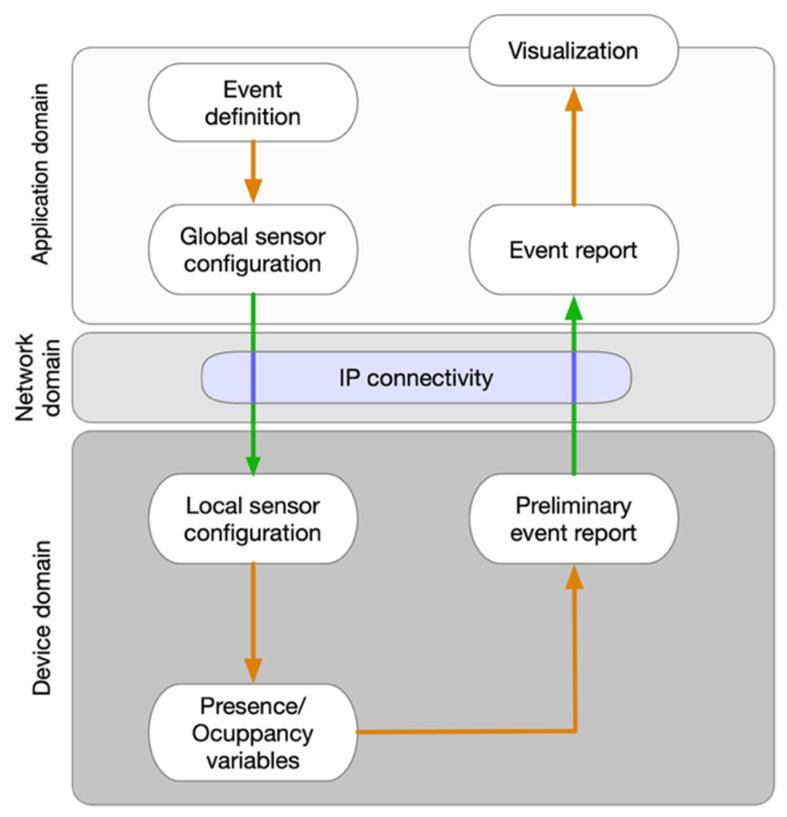
System architecture overview and information flow.

As a result of the system specification and requirements outlined in the previous section, a three-tier architecture is proposed to support the underlying system. This architecture aligns with the latest IoT standards and architectures (IEEE P2413, ETSI M2M-OneM2M, etc.) [42,43] where each tier corresponds to an operation domain of the system. Thus, the first tier focuses on sensing and data acquisition, i.e., the device domain; the second on networking and data communication, i.e., network domain; and the third on service delivery and orchestration, i.e., application domain. Figure 1 shows the proposed system architecture and information flow.

As it can be observed in Figure 1, the definition of an event generates a global configuration of Wi-Fi sensors that is used to assess the occupancy of the event environment. The event definition consists of a date, start and end time of the measurement, as well as the identification name of the event. This configuration is broadcasted to all Wi-Fi sensors involved in the assessment, and each sensor adapts its local configuration with the new received commands.

Once an assessment associated with an event is finished, all Wi-Fi sensors involved clean the gathered data and prepare it to be sent to the server. The server is in charge of merging all data received from Wi-Fi sensors related to the same event and performing an in-depth analysis of them. Finally, the server prepares the data for visualization by creating graphs and reports.

**Figure 2:**
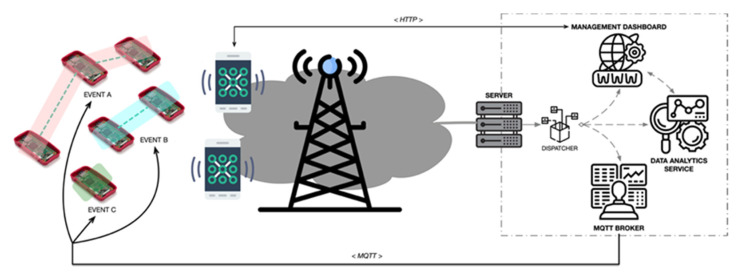
System overview.

Each one of the operational domains of the proposed architecture is detailed below. Figure 2 shows the system overview and the interaction between each element. As it is shown, the system interactions are based on the use of wireless communications through the network domain. Specifically, the users of the system are able to communicate with the application domain through HTTP, using any web-enabled device, i.e., smartphones, tablets, PCs, etc., using an internet-based connection. These interactions are required to configure and manage the Wi-Fi sensor and also to obtain the results of the event’s monitoring. The Wi-Fi sensors, on the other hand, communicate with the application domain using the MQTT (Message Queueing Telemetry Transport) protocol, one of the most used protocols for IoT device communications. The interaction between sensors and the application domain has as a goal the exchange of management and configuration information and the delivery of the data monitored by each sensor. Given that the Wi-Fi sensors are not able to connect directly to the internet, the connection between the device domain and the application domain is carried out using the operator’s device (smartphone) as an intermediary, acting as a Wi-Fi access point for the Wi-Fi sensor.

### 4.1. Device Domain

**Figure 3:**
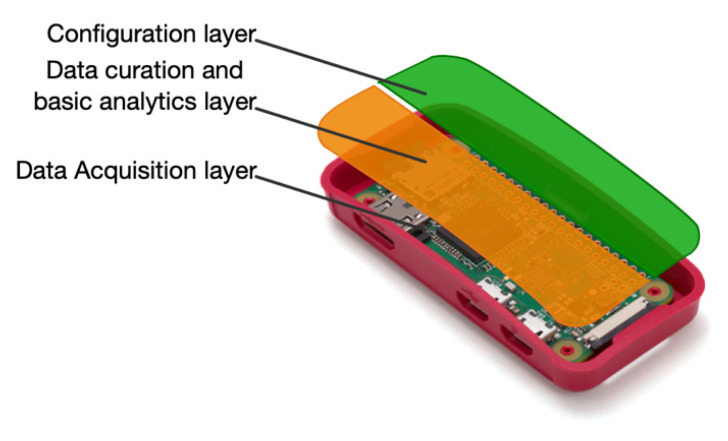
Operation layers of the Wi-Fi sensors at the device domain.

The device domain is made up of Wi-Fi sensors capable of capturing Wi-Fi probe requests issued by any other Wi-Fi device. These Wi-Fi sensors can operate individually or collectively and are composed of three layers of operation; Figure 3 shows one dedicated to managing the configuration of measurements, another to clean the sensed data, and a low-level one focused on capturing Wi-Fi probe requests. This approach facilitates independence between layers, allowing for updating or substituting any of them easily.

**Figure 4:**
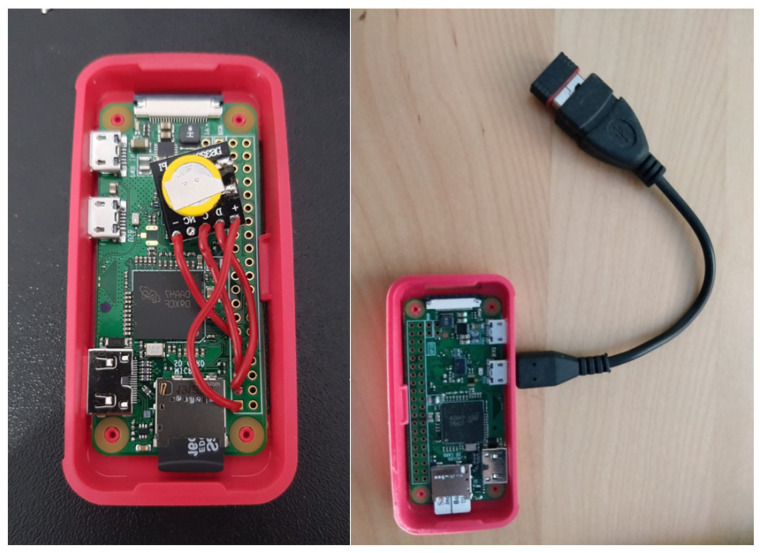
Wi-Fi sensor prototype showing the RTC clock (left) and the external Wi-Fi adapter (right).

Each Wi-Fi sensor is based on a Raspberry Pi Zero W (Raspberry Pi Foundation, Cambridge, UK) single board. These boards are provided with a wireless interface and Bluetooth connectivity. In addition, an additional wireless network adapter was added (TB Ralink 5370; MediaTek Inc., Hsinchu, TW). This way, each sensor can be connected to a Wi-Fi LAN and monitor Wi-Fi probes at the same time. Finally, a modification was applied to the Raspberry equipment that aims to solve the problem identified in the study carried out in [37], which consists of the integration and use of external clocks RTC DS3231 to obtain timestamps for the data collected, avoiding data inconsistencies when being processed by the server. In this way, duplicate data are avoided, and the correlation of information captured by different sensors is facilitated. Figure 4 shows a real example of the Wi-Fi sensors implemented.

#### 4.1.1. Data Acquisition Layer

The data acquisition layer is based on a C program (airodump-ng tool from aircrack-ng suite [44]) that interacts with the driver of the Wi-Fi USB dongle capturing the IEEE 802.11 radio frames and analyzing its header on the fly. Within the header, first, the MAC address of the station is analyzed in order to determine if it is a real MAC address (registered by the IEEE) or a randomized one (detailed explanation is presented in Section 4.1.3). These are increasingly used in probe request messages in order to defeat Wi-Fi tracking systems. In order to capture the highest possible number of frames, the data acquisition layer software performs periodic sweeps between channels (2.4GHz and 5GHz bands). This feature allows the program to obtain frames from devices operating in any channel.

**Figure 5:**
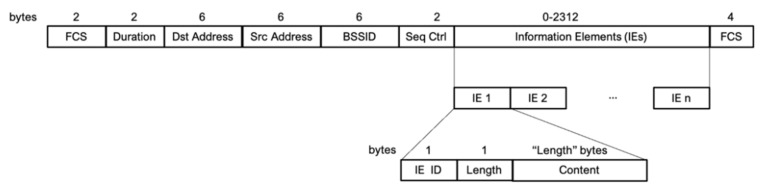
IEEE 802.11 probe request frame format.

**Figure 6:**
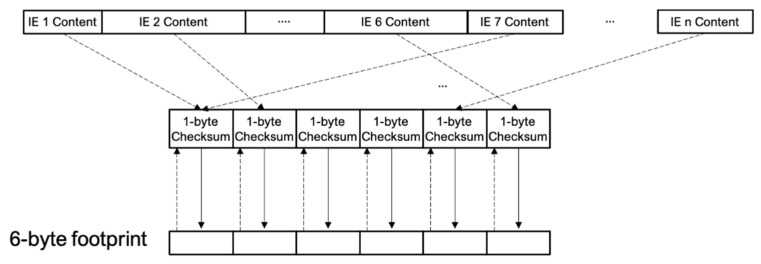
Procedure for obtaining the 6-byte footprint.

In the case of real MAC addresses, we used it as a unique device identifier because it is always unique. In the case of random MAC addresses, we analyzed the information element (IE) fields of the IEEE 802.11 frame and generated a 6-byte footprint as a unique identifier. The footprint is generated in the following way:

First, a 6-byte footprint is initialized to zero (00: 00: 00: 00: 00: 00). The IEs of each Wi-Fi probe are analyzed, as shown in Figure 5. Once the IEs of the Wi-Fi probe are checked (avoiding using the ESSID, since it is always present and can be highly variable due to the fact that the same device can ask for several networks in a small period of time), a mini footprint is calculated. This mini footprint is a 1-byte value calculated as the checksum of the bytes contained in the analyzed IE. For those IEs that present highly variable values between probes issued from the same device, e.g., IE_DS_PARAM or IR_EXT_CAP, the mini footprint is calculated as the code of the IE itself (0×03 for IE_DS_PARAM, 0×7F for IE_EXT_CAP, etc.). Sequentially, each time a mini footprint is obtained, its value is added to that of the content of the next byte of the footprint, and the mask 0xFF is applied to it. If the number of mini footprints is greater than 6, it begins again with the first element of the footprint, and so on. This process is summarized in Figure 6.

In this way, as the unique identifier has the same length and shape as a MAC address, all devices are uniquely identified by a 6-byte code that is either based on its real MAC address or the IE footprint of its probe requests. In this sense, all sent data are anonymized by transforming the real MAC addresses, as well as randomized MACs, sent to the application domain into a 6-byte unique device user identification (UUI), following the previous work detailed in [45,46].

As mentioned before, the data are sent using MQTT as the transmission protocol, formatting the data-like messages, and publishing in pre-configured queues. Additionally, the messages are just used for the data analysis process, and then they are erased to avoid maintaining the captured information in the server.

The data collection process is controlled by a set of configuration variables stored in a configuration file within each sensor. These variables specify, among other things, the observation time and the frequency of sending data to the server. By default, each sensor gathers the identifiers observed every 15 min, stores the information locally in the sensor, and once the measurement event is finished, all the monitored information is then sent to the server for further processing.

During the gathering process, the data are stored in a set of files in the sensor, depending on the type of information and how it is classified. These files, in CSV (comma-separated values) format, are classified into the following:

- Probe files: containing information about each captured probe during the event.
- Statistics files: containing summarized information about each MAC address seen in the probes.

Table 1 shows the information stored in each file and a brief description of the fields in the corresponding CSV.

#### 4.1.2. Configuration Layer

The configuration layer determines the specific behavior of the sensor for each event. When one or more sensors are assigned to an event, a record is generated and stored by the system for each tuple (*event_id, sensor_id*). Furthermore, the system generates a configuration file (CSV file) for each affected sensor. This configuration file contains one line of text for each event that this sensor must operate. These configuration files are generated when a new record of measurement is created at the server.

Thus, prior to the event, each sensor to be used receives an updated file via MQTT containing the information related to the assigned events. For each event, the sensor will receive a code, an alias, and start/end dates.

The file is kept stored in each sensor, and there is a specific scheduler software to monitor the file contents in order to start and stop the data gathering process, according to the configured events. A new scheduling configuration file can be sent at any time to modify future events or add new events to a specific sensor.

The configuration data for each event are associated with the data obtained by the sensor. This allows easy identification of data in the server during the data analysis process.

#### 4.1.3. Preprocessing and Basic Analytics Layer

While the sensor is monitoring and gathering information, there is an initial preprocessing and analytics process being run in the sensor. This part of the system is aimed to determine which captured MAC addresses are the actual MAC address of the device or a random MAC used for probing. This is a crucial step in the accurate calculation of the number of physical devices present in the event, as a single device might send probes using different MAC addresses.

Up to four different types of MAC addresses can be classified in the captured probes, depending on if they are real or random addresses. The classifications we used for the data files in the Wi-Fi sensors are the following:

- MAC address lookup (MAL) addresses: real device-dependent MAC addresses, registered with a manufacturer OUI.
- Local MAC authentication (LMA) addresses: random locally generated MAC addresses. They can be identified because the second bit in the first byte of the address is set to one.
- Company ID (CID) addresses: random generated MAC addresses that are registered by a manufacturer and therefore use a specific OUI. These can be seen as a subset of the LMA addresses.
- N/A addresses: unknown unregistered MAC addresses. These cannot be classified into the other groups as they are not registered or identified as local random addresses.

For our preprocessing purposes, we determined that any MAL address captured can be identified as an actual device, while the other three types of addresses might not correspond to a device one to one, given that a single device will probably send probes using various random MAC addresses.

In order to determine which of the MAC addresses belong to the same physical device, we developed a probe analysis process. This process relies on the generation of a footprint for each probe captured, based on the information obtained in specific fields (IEs) of the captured probes. The footprint is basically a set of bytes generated using information from different fields in the probe and its length. By selecting this information carefully, we were able to deduce a set of bytes that would be similar in all the probes coming from the same physical device, even if the source MAC address was different.

The calculated footprints were stored along with other information for the probes (see Table 1), and later they were used for the creation of a list of possible physical devices. For each unique real MAC address captured in the probes, an entry is created in that list, using a one-way hash function applied to the real MAC as an identifier [45]. For random MACs, only one entry is generated for all of them that share the same footprint, using, in this case, the footprint as an identifier. For consistency purposes, footprints are represented using 6 bytes separated by colons. Counting the entries on this list allows for an accurate estimation of the number of single physical devices present at an event at a certain interval of time. We treated the real MAC addresses differently from the random MACs because we assumed that most devices will not send both types of addresses in their probes. This faulty behavior was identified in [36] but has since been fixed in operative system updates [47].

### 4.2. Network Domain

The communication between the device and the application domains is carried out by a second Wi-Fi interface, which is embedded in the Raspberry Pi board. The device was configured so that it tries to connect to an internet access point (internet AP) with a specific ESSID. This internet AP can be deployed ad hoc using a smartphone when communication between the device and the server is required. The main tasks that require internet access from the sensor are when it needs to obtain the calendar of events from the server, when the operator wants to check the status of the device, and for sending the data to the server after the event has finished.

As we already stated, the communication between the device and the server was based on the MQTT Protocol. MQTT is a publishing/subscription protocol widely used in IoT environments due to its simplicity, lightness, and robustness. It is based on the use of a centralized broker, which defines a series of message queues that act as communication channels between clients connected to it. The clients can act as publishers (generating messages for a certain queue), subscribers (consuming the messages from a queue), or both at the same time.

In our network domain, each Wi-Fi sensor acts as an MQTT client able to subscribe to configuration queues and to publish to data queues. Queues are defined in the broker and are identified by hierarchical labels called topics.

The sensor publishes messages on MQTT topics for reporting its status (e.g., measuring or in standby) as well as the files with the collected data. In addition, it also subscribes to MQTT topics so it can be remotely configured (for updating the calendar of events), requests a reset in case of malfunctioning, or to perform a software update.

We used the hierarchical nature of topics to automatically subscribe the Wi-Fi sensor to specific queues (meaning that it is possible to select a single sensor to send a message) that can also be accessed using wildcard-based topics (e.g., we can send a single configuration file to a sensor using a CONFIG/DEV_ID queue, but we can also send a configuration file to multiple sensors using CONFG/# as the queue identifier).

We deployed the MQTT broker in our server, along with the application domain, which will be covered in the next section of this paper. We selected the open-source Mosquitto broker implementation [48] as a broker. Wi-Fi sensors must gain internet access prior to being able to use the specific message queues, for which they can use any device that could be configured as a wireless access point, namely, the smartphone of the operator at the event. All MQTT communications are secured by using it through TLS.

Although they are deployed in a single hardware sensor, the web application in charge of managing the data collected during events receives the data from the broker acting also as an MQTT client, subscribed to the data queues defined in the broker, and sends new configuration messages or software updates using the same mechanism.

### 4.3. Application Domain

The application domain was designed to perform three tasks: event management, Wi-Fi sensor management, and heavy data analysis (collect and merge data of an event from different Wi-Fi sensors, data visualization, etc.). As we stated before, data collected by Wi-Fi sensors is sent via secure MQTT to the server, which runs a broker. A programable MQTT client located in that server subscribes to MQTT topics published by the Wi-Fi sensors and performs specific actions defined in terms of bash scripts. In the case of the MQTT messages conveying data, the corresponding script oversees storing the results in the file system following a directory structure aligned with the MQTT topic hierarchy, which includes in its path the unique identifier of each Wi-Fi sensor and the gathering period (15 min by default) to which the data correspond. For convenience, the unique identifier was the MAC address of the internal Wi-Fi chipset of the Raspberry Pi. The gathering period was identified by a string with the format YYYYMMDDhhmm that indicates the start of the gathering period (for example, 202007122115 indicates the 15 min period from 21:15 to 21:30 of July 12th, 2020). This naming convention was very handy for the storage (and later retrieval) of the data in the file system. As we said, the directories follow the same structure used for the definition of hierarchical MQTT topics, so, for example, the list of Wi-Fi stations detected during the period 202007122115 by a device with MAC address b8:27:eb:5a:cf:77 is published on the topic *WIFI/b8:27:eb:5a:cf:77/STA/202007122115.STA*, which is stored by the MQTT client in the *~/prj/res/WIFI/b8:27:eb:5a:cf:77/STA/202007122115.STA* file path.

If the configuration file is not stated otherwise, after a calendar event is finished and all of its associated data files are sent by the Wi-Fi sensor, a specific MQTT message CAL_PROC is published so that the server can proceed with the data analysis. This ends in a series of CSV files stored in the server’s file system and can be later accessed by the web application running in it to visualize the results.

The web application allows the definition of events, the assignment of Wi-Fi sensors to events, and the visualization of results after the events finished. The web server is based on node.js [49] and its web framework, Express [50]. It also uses the SQLite database for managing the information related to events, Wi-Fi sensors, and user of the system credentials. It was developed following a RESTful approach [51]. That means that the functionality offered by the server was encapsulated into web resources and offered by a RESTful API to other components of the platform.

**Figure 7:**
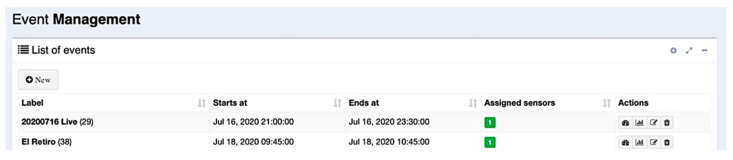
Event list view in the web application.

**Figure 8:**
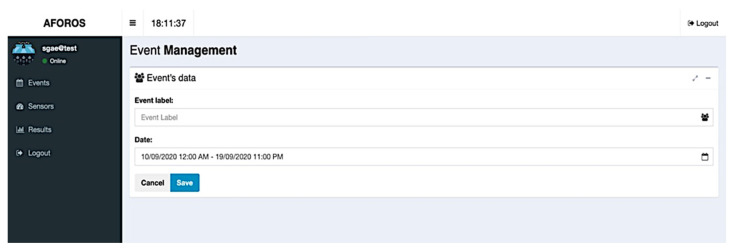
Events edit form in the web application.

User–server interaction is essentially carried out through the web application, allowing easy event management, sensor provisioning, and data visualization. In Figure 7 and Figure 8, it is possible to see the main views regarding event management in the web application. The first figure shows the view that lists the current events in the server database, including their starting and ending dates and the Wi-Fi sensors assigned to them. For each one of these events, it is possible to edit their information, or it is possible to add new events using the edit form as shown in Figure 8.

**Figure 9:**
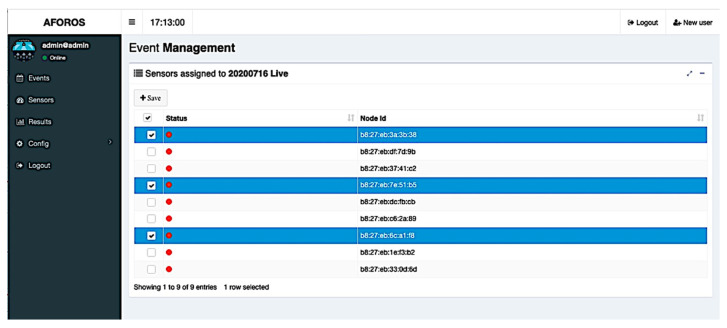
Sensor assignment in the web application.

Assigning Wi-Fi sensors to events is also a task that can be carried out using the web application. This action can be started from the event’s list (seen in Figure 7). One or more Wi-Fi sensors can be assigned to each event, selecting them from a list of available sensors, as seen in Figure 9. In that list, it is possible to view the current status of each sensor (that is, if the Wi-Fi sensors are connected and available at that moment).

**Figure 10:**
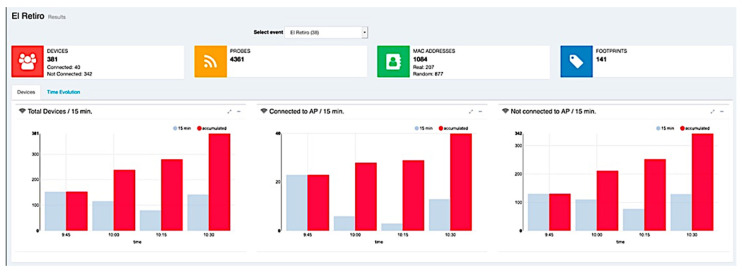
Graphical view of the occupancy of a space where an event is organized.

**Figure 11:**
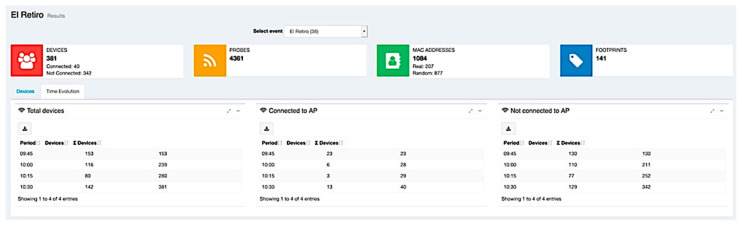
Information presented by the web application about the occupancy of a space where an event is organized.

Finally, as we already pointed out, the web application was used as a data visualization tool, allowing the graphical visualization of the calculated attendance in each event (see Figure 10) and more detailed information about the event in time intervals (Figure 11).

Additional to the MQTT broker and the web application, the server is composed of other software modules in charge of performing an additional analysis and data processing to clean and present the information. These modules are essentially scripts and binary compiled programs that carry out tasks such as copying and formatting the CSV data, monitor new data obtained through the MQTT client, determine the status of the sensors, etc. Among these tasks, there is a very important one, which allows the aggregation of data from multiple Wi-Fi sensors if they provide data from the same event. This program can find duplicated data from different files by using the UUI and generates a single information source to be presented through the web application. Since several Wi-Fi sensors can detect the same UUI (that is, a device), the server must eliminate duplicated UUIs related to the same event.

## 5. Cases of Study and Validation

The validation of the proposed system was carried out through four experiments. Before conducting these experiments, we performed some in lab tests in an isolated facility (without external uncontrolled and unexpected Wi-Fi signals). During this initial testing, we could corroborate our system to identify every Wi-Fi device uniquely. To perform this test, we used seven smartphones with Android 9 and 10 and a Wi-Fi sensor running for 15 min and static in the middle of the facility. After finishing this initial test, we started the validation process based on two different experimental phases.

In the first one, we defined two case studies in controlled environments, and the second one was composed of two field experiments. The preliminary case studies were intended to allow us to fit the early basic analysis performed by the Wi-Fi sensors and adjust the usability and user experience. An important difference between the case studies and the field experiments is that the first ones were carried out by the authors in controlled spaces while the latter were carried out by third parties and without the direct control of the authors. These experiments and the results obtained in each one are detailed below.

### 5.1. Preliminary and Tuning Experimentation

As we stated before, these initial experiments were oriented to adjust the detection of mobile devices in an area of controlled movement, as well as to make some adjustments to the final visualization web application. In this sense, three tests were carried out, and the setups are detailed in Table 2.

The first preliminary experiment (prel_01) was carried out at a gas station. A sensor was used here, which was located at the entrance to the premises. The total number of people who visited this environment during the experiment was recorded by hand by the researcher in charge. A second preliminary experiment (prel_02) was conducted at a public space reserved for vehicle inspection (MOT). Again, the total number of people who visited this space was controlled by hand. The results obtained in each preliminary study are detailed in Table 3.

The results show that the estimates made by the system in these preliminary tests were realistic yet not as close to the observed reality as expected, pointing out that some adjustments had to be made in our system to increase its accuracy. The total number of devices is calculated as a sum between the UUI obtained from real MACs and the footprints, which, as indicated in the previous sections, are virtual identifiers obtained from the random MACs sent by the devices. In this sense, and always assuming one person per device, for the first experiment (prel_01), we had an expected number of people of 25, and the system detected 21 real MACs and 15 footprints. The deviation between the detected result and the expected one was corrected by adjusting the calculation of the 6-byte footprints. This adjustment was focused on the analysis of all IEs to determine those that varied between probes sent by the same device, e.g., IE_DS_SET or IE_EXT_CAP. Using these IEs in the calculation of the 6-byte footprint resulted in different values for the same device, overestimating the final number of people. Finally, and as explained in Section 4.1.1., for these variable IEs, we decided to use their code instead of their value.

Finally, after the adjustments in the footprint calculation algorithm, the second experiment (prel_02) was carried out. In this case, we counted 105 people but detected 34 real MACs and 76 footprints, giving us a total estimation of 110. An error in the CSV reading system was identified and corrected after this experiment, so the actual estimation should be lower and closer to the real expected result.

### 5.2. Validation

The validation of the system, beyond the first preliminary studies that helped us to tune and correct it, was carried out by means of two experiments in real environments, where actual events were taking place. The details of these experiments are shown in Table 4. Both experiments were based on measurements of attendance at events in open and closed spaces and with limited and controlled capacity. To maintain the anonymity of the places and events used for this validation, their name and location are omitted.

The first experiment (real_01) was carried out during an outdoor show lasting 3 h. The maximum capacity of the space reserved for the show was 800 people, all seated around tables with a separation of 2 m between them and between the assistants at each table. The event took place in a non-residential area. In addition, this event was held on a Saturday in July 2020, at night. For its part, the second experiment (real_02) was carried out in a closed concert hall, with a maximum capacity of 500 people. This space is in a residential area, with night shops and restaurants nearby. For both experiments, we used a single static Wi-Fi sensor located at a specific point of the enclosure, which was not the geometric center in either case. For comparison purposes, we were able to access the official register of attendees, as counted by the event organizer. This allowed us to perform a more exhaustive analysis of the results obtained. These results are summarized in Table 5.

The results shown in Table 5 indicate that in both experiments, the system was able to determine the occupancy of the event with a margin of error of less than 4%. The event used as the basis of the experiment real_01 had a final attendance of 700 people, 100 less than the maximum allowed. The system, in this case, estimated the attendance of 677 people, 3.29% less. For its part, the event measured in the real_02 experiment had an attendance of 382 people as public, 40 employees of the venue, and 20 guests by the room and the artist. In this case, the system estimated the presence of 435 people, 1.58% less.

## 6. Discussion and Conclusions

In this paper, we presented a complete attendance measurement system based on Wi-Fi sensors. The aim of our proposal was to be able to make an estimation of actual occupation in public events or spaces without relying on complex counting systems or installed infrastructures that are not always feasible in all environments. Moreover, one of the main advantages of this type of sensor-based ad hoc system is that it does not require any interaction with the attendants or the event’s organization, making it an unobtrusive and especially discreet way of counting people.

Our system was based on using a small sensor with Wi-Fi tracking capabilities. These sensors can be powered by a battery and carried around through the venue during an event. We count on that, nowadays, most, if not all, attendants to public events carry at least one Wi-Fi-enabled device (smartphones, smartwatches, etc.). These devices are continuously sending probes so they can find nearby access points, and our sensors take advantage of that, capturing and monitoring that kind of traffic.

Ideally, each device would send its own MAC address to identify itself, but this is not the case at all in modern devices, as we explained before. In fact, most probes captured used randomized MAC addresses, which make it difficult to map probes and devices. Determining the number of physical devices using the probe information has proven to be one of the main challenges in our design. To overcome this obstacle, we propose a footprint-based mechanism, generating an identifier for probes based not only on MAC addresses but on other information elements present in probes. Combining various sources of information to generate the footprint byte string identifier allowed us to significantly increase the accuracy of the estimations, as we observed that probes with the same physical origin generate the same footprint. In the case of real MACs, the privacy of device owners is guaranteed by an anonymization process where a real MAC is transformed into a unique ID, the UUI.

Our proposed system is not only composed of isolated sensors. In fact, we designed and developed a complete architecture for our sensors, composed of the Wi-Fi sensors themselves and a server accessed via the internet. The sensors communicate with the server using the MQTT protocol, but they do not need continuous communication to work properly. They can connect to access points in the operator’s smartphone to receive configurations and send the gathered data. Additionally, the server performs processing tasks and is equipped with a web application that allows the management of Wi-Fi sensors and the estimations presentation. Another key point of this architecture is the possibility of configuring more than one sensor for a single event, making the system more scalable and allowing it to obtain more information than using just one sensor per event. To enable this multi-sensor network configuration, it was necessary to add a real-time clock module to each Wi-Fi sensor. This, together with processing on the server of the data received from each Wi-Fi sensor, make it possible to merge and clean the data from different sensors for the same event.

Regarding the validation of the system, it was carried out in two phases. The first one aimed to test the correct behavior of the system and allow us to tune and modify our algorithms to make them more accurate. The results of these preliminary tests showed some errors and some needed adjustments in the counting system but validated its feasibility and that the attendance estimations were close to reality. In the second phase, we validated the system by testing it in real-life events without the authors’ monitoring. The results obtained in these experiments showed closeness between the actual attendance and our estimation, with an error below 4% in both experiments.

The analysis of these results, together with the characteristics of the experimentation space and the configuration of the Wi-Fi sensors, indicate that it is possible to improve this margin of error. The system allows the inclusion of several Wi-Fi sensors for the analysis of the same event, as well as its movement throughout the venue, but this characteristic could not be used in those experiments due to current limitations in public events. In addition, the Wi-Fi sensors are configured to do sequential sweeps on all possible Wi-Fi channels. This could lead to missing out on possible devices that operate for a short period of time on a certain channel.

Overall, although we consider these results good and promising, we plan to take further actions to improve their accuracy. For example, the difference between the number of devices and people is not a parameter that Wi-Fi sensors can easily measure. To mitigate this, we considered the concept of “the ratio of actual people vs. devices detected”, assuming it would be a number close to 1 (a Wi-Fi-enabled device for each person). Of course, this ratio has some error margin due to several issues (people carrying several Wi-Fi devices, the same devices sending real and randomized MAC addresses simultaneously, etc.). In this sense, we thought this ratio could be adjusted by analyzing current statistics about use and adoption of smartphones and wearables, as well as by performing some surveys in different scenarios where this system can be applied. Therefore, this ratio will be added as a parameter to be adjusted at any time to improve the solution and its estimations.

Moreover, we plan to carry out further experiments as soon as possible, using more than one sensor to measure the event and moving the sensors around the venue during its duration. Using those results, we will try to further improve our calculation algorithms, improving the footprint generation and using more data per event. Additionally, considering new parameters and correction factors, such as the type of the event, where the event takes place, etc., could improve the final estimation.

Another future work to be carried out is to study the scalability limitations of our sensor system. We plan to test the sensors in massive attendance events to find out the maximum number of probes that a single Wi-Fi sensor could capture in these environments and determine how many sensors should be used and how they should be to improve the accuracy of estimations.

## Figures and Tables

**Table 1:** Fields of the CSV files generated from captured probes.

File	Field	Description	Example
**Probe files**	Timestamp	Timestamp for the captured probe	*20200714161453.596*
	Type	Type of MAC address (see Section 4.1.3)	*MAL*
	Channel	Channel used to transmit the probe	*7*
	Power	Received power (dBm)	*−80*
	Footprint	MAC-like set of 6 bytes generated from probe information	*25:3E:12:FF:5D:4E*
	Numies	Number of IEs	*8*
	Ieseq	The sequence of IE codes	*01:32:03:2D:7F:6B:DD*
	Ielens	Length of each IE in hexadecimal	*04:08:01:26:08:07:11*
	Ievals	Values of each IE in hexadecimal	*27:9E:06:86:C0:09:18*
	Oui	Organization identifier obtained from the MAC address	*apple*
	Essid	Identifier of the network connected (if available)	*MYSSD_AF8A*
	Userid	Unique identifier derived from MAC address or footprint generated from the probe information	*b477fgj324418fgbZn*
**Statistic files**	Userid	Unique identifier derived from MAC address or footprint generated from the probe information	*477fgj324418fgbZn*
	Minact	The appearance of UUI in a sampling period (15 min by default)	*111110110001011*
	Pwr	Received power (dBm)	*−80*
	Oui	Organization identifier obtained from the MAC address	*apple*
	Type	Type of MAC address	*MAL*
	AP	MAC address of the AP connected (if connected)	*70:F8:2B:11:4A:C9*
	Essid	Identifier of the network connected (if available)	*MYSSD_34D5*

**Table 2:** Description of experiment setups.

ID Test	Location	Covered Area	Duration	Sensor Movement
Prel_01	Gas station	3793.59 m^2^	15 min	No
Prel_02	MOT	1746.52 m^2^	30 min	No

**Table 3:** Preliminary experiments’ results.

ID Test	Probes	MAC Addresses (Real/Random)	Footprints	Devices	People
Prel_01	876	117 (21/96)	15	36	25
Prel_02	1882	311 (34/277)	76	110	105
Prel_03	11,238	2205 (56/2159)	76	132	70

**Table 4:** Validation experiments’ results.

Id Experiment	Outdoor	Covered Area	Duration	Capacity	Sensor Movement
Real_01	Yes	2000 m^2^	180 min	800	no
Real_02	No	1500 m^2^	120 min	500	no

**Table 5:** Validation experiments’ results.

Id Experiment	Probes	Mac Addresses (Real/Random)	Footprints	Devices	People
Real_01	58,734	26664 (442/26222)	236	677	700
Real_02	37,562	17552 (302/17250)	133	435	442

## Data Availability

Not applicable.
